# Efficient implementation of a real-time estimation system for thalamocortical hidden Parkinsonian properties

**DOI:** 10.1038/srep40152

**Published:** 2017-01-09

**Authors:** Shuangming Yang, Bin Deng, Jiang Wang, Huiyan Li, Chen Liu, Chris Fietkiewicz, Kenneth A. Loparo

**Affiliations:** 1 School of Electrical Engineering and Automation, Tianjin University, 300072, Tianjin, China; 2 School of Automation and Electrical Engineering, Tianjin University of Technology and Educations, 300222, Tianjin, China; 3 Department of Electrical Engineering and Computer Science, Case Western Reserve University, 44106, Cleveland, Ohio, USA

## Abstract

Real-time estimation of dynamical characteristics of thalamocortical cells, such as dynamics of ion channels and membrane potentials, is useful and essential in the study of the thalamus in Parkinsonian state. However, measuring the dynamical properties of ion channels is extremely challenging experimentally and even impossible in clinical applications. This paper presents and evaluates a real-time estimation system for thalamocortical hidden properties. For the sake of efficiency, we use a field programmable gate array for strictly hardware-based computation and algorithm optimization. In the proposed system, the FPGA-based unscented Kalman filter is implemented into a conductance-based TC neuron model. Since the complexity of TC neuron model restrains its hardware implementation in parallel structure, a cost efficient model is proposed to reduce the resource cost while retaining the relevant ionic dynamics. Experimental results demonstrate the real-time capability to estimate thalamocortical hidden properties with high precision under both normal and Parkinsonian states. While it is applied to estimate the hidden properties of the thalamus and explore the mechanism of the Parkinsonian state, the proposed method can be useful in the dynamic clamp technique of the electrophysiological experiments, the neural control engineering and brain-machine interface studies.

Parkinson’s disease (PD) is a kind of neurodegenerative disease characterized by the degradation of substantia nigra dopaminergic neurons^1^,^2^,^3^,^4^, and the cellular mechanisms inducing neuronal death are still unknown. The most significant symptoms are movement disorders such as shaking, slowness of movement, rigidity and problems with walking and gait. They are closely related with the loss of the ability of thalamocortical (TC) neuron to relay the excitatory sensorimotor cortical information. In fact, the loss of such ability is caused by the low-frequency pathological rebound bursts in TC neurons^5^,^6^,^7^,^8^,^9^. In a TC neuron, low-threshold T-type calcium current is essential to the generation of the rebound bursts induced by the excessive γ-Aminobutyratergic projections from the basal ganglia^10^. The dynamics of the low-threshold T-type calcium current are considered as the thalamocortical hidden properties. Besides, the thalamus is a vital gateway to the neocortex in which sensory pathways to the cortex go through appropriate thalamic nuclei^11^,^12^,^13^,^14^.

Previous experimental paradigms have been proposed to explore the functions of thalamus under the neurodegenerative state of movement disorders. At the cellular and microcircuit levels, intracellular and extracellular recording techniques including voltage clamp and dynamic clamp are widely applied in electrophysiological experiments^15^,^16^. In comparison with voltage-clamp techniques, the dynamic clamp technique can verify more sophisticated hypothesis in electrically excitable neurons. It has been used to apply conductance to the neurons to investigate the mechanism underlying rhythmical bursts, which is useful in the research of PD^17^. Besides, researchers have used the dynamic clamp in TC neurons *in vitro* to explore the effects of the ionic current on bursting activities under the Parkinsonian state^18^. As a result, the dynamic clamp technique is useful in the research of the Parkinsonian mechanism. The dynamic clamp technique uses the measured membrane potential to control the amount of current injected into a neuron. Because the membrane potential changes faster than the hidden variables, the amount of the injection current is higher and changes fast. It would hurt the neuronal physiological structure and cost more energy. Therefore, estimating the hidden variables in a neuron can assist dynamic clamping. However, there remain two challenges that would limit the performance and application of dynamic clamp techniques in the research of the movement disorders. Firstly, the critical hidden properties underlying the membrane potential for the investigation of the pathological states cannot be observed directly by current electrophysiological dynamic clamp techniques. Previous studies have revealed that the dysrhythmia of PD is generated by T-type calcium channel deinactivation in the Parkinsonian thalamus that is reflected by the variations of the concentration for T-type calcium ions in the TC relay neurons^6^,^19^,^20^,^21^. The prediction of the slow variable in the neuron model is also essential in the neural control engineering (NCE) and brain-machine interface (BMI) projects^22^,^23^,^24^. Secondly, hardware-based dynamic clamp systems are limited by their poor programmability, while software-based systems cannot guarantee the real-time performance^25^,^26^. As a result, there exists a demand for a novel approach with the advantages of high computational efficiency as well as programmability to remedy the disadvantages of the conventional implementations.

During the past decades, the unscented Kalman Filter (UKF) has been used as an efficient method for the dynamical estimation of the nonlinear systems^27^,^28^,^29^. Previous studies have used the UKF algorithm in the estimation of spatiotemporal cortical dynamics^30^,^31^. Considering the importance of thalamus on the investigation of PD, it is useful to estimate the thalamocortical ionic state parameters from the membrane potentials contaminated with noise. However, the UKF has shortcomings when applied to TC dynamical estimation. The states of the ion channels change rapidly during the process of external stimulation, which requires the latency of the UKF to satisfy the demand of real-time dynamical tracking constraints. Moreover, the UKF algorithm is highly complex and involves a large volume of data and for each iteration, numerous matrix multiplications and inversion operations are performed. These factors limit the UKF in physiological applications.

Aiming at speeding up the on-line computational performance of the UKF, a hardware implementation with high computational capacity is required. The complex computation is a challenge for a fixed-point processor system, especially for the implementation on a hardware chip. Manifesting the advantages of low energy consumption, high reliability, parallel processing and fast time to market^32^,^33^,^34^,^35^,^36^, the Field Programmable Gate Array (FPGA)-based implementation shows promise for a neural estimation system with higher performance. In order to estimate the experimentally inaccessible dynamics of the neuron, the UKF algorithm is required to be implemented into the neuron model^23^,^24^,^31^. As a result, another challenge in the neural dynamical estimation is the implementation of the nonlinear neuron model to reconstruct unobserved intracellular variables and parameters only from measured membrane potentials. Biologically conductance-based computational model of the TC neuron has a number of nonlinear functions with multiplications and sigmoid functions, which cannot be used in the parallel-structured implementation of UKF algorithm. The optimization of this model is necessary for the implementation of the proposed estimation system.

In this paper, we present a real-time thalamocortical dynamical estimation (RTDE) system to explore the mechanisms of the movement disorders by estimating the hidden properties in a noisy measurement environment with an improved computational performance. The system receives the membrane potentials of the TC neuron model and outputs the estimation results of the hidden properties. Since the UKF system needs to be applied into a conductance-based TC neuron model, a cost efficient TC (CETC) neuron model is proposed, which is implemented with low hardware overhead to achieve real-time execution. To the best of our knowledge, no previous works have proposed a real-time system for TC dynamical estimation of the ion channels. Our study is certainly of interest because it facilitates the explorations of the mechanisms of the Parkinsonian state, and the performance enhancement of the current electrophysiological technique such as dynamic clamp technique. The general scheme of the proposed work, targeting the estimation of the pathological states, can be applied in the studies based on the dynamic clamp technique. The proposed system can be also useful for the performance improvement of neuromodulation and investigation for the mechanisms of kinds of diseases including Huntington’s disease, epilepsy and Alzheimer’s disease^37^, ^38^, ^39^, ^40^. The proposed study is the first prototype of a hardware-based platform that uses a real-time neural dynamical estimation system to track the biological characteristics of the thalamus underlying the neural firings. This work provides a new perspective for neural engineering, and can be further applied in NCE and BMI projects.

## Results

### System description

**Figure 1:**
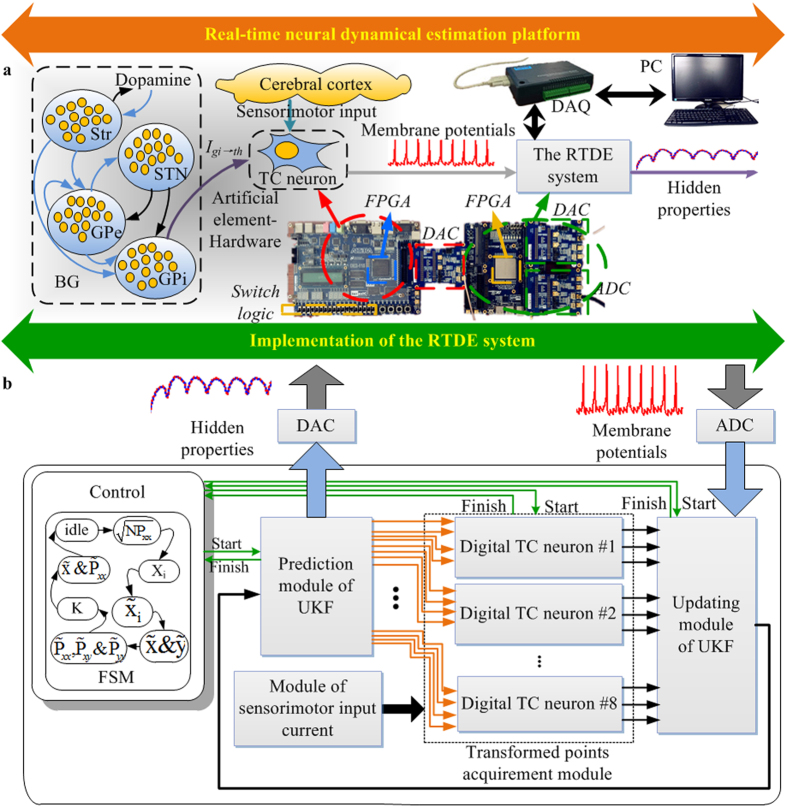
Proposed hardware platform for TC dynamics estimation. Schematic descriptions and images of components for the proposed hardware platform with the RTDE system. (**a**) Real-time neural dynamical estimation platform. (**b**) The implementation of the RTDE system.

A schematic diagram of the experimental setup and the general overview of the electronic system are proposed in Fig. 1. This platform is established to evaluate the performance of the proposed RTDE system. The RTDE system is equipped with analog-to-digital conversion (ADC) device to receive the input signals of the thalamocortical membrane potentials and digital-to-analog conversion (DAC) device to output the analog estimation results of the hidden properties to the oscilloscope or the DAQ device. The analog outputs can be also acquired by a data acquisition (DAQ) device, which will be visualized on a personal computer or used in neuromodulation systems.

The proposed hardware implementation of the RTDE system is divided into three parts, which are the prediction module of UKF, the transformed points acquirement module and the updating module of UKF. The detailed description of each module is proposed in the following sections. In terms of control logic of the RTDE system, a finite-state machine (FSM) is used as a block of combinational logic that determines the state transition. The cortical excitatory input current from the brain sensorimotor is implemented in the module of sensorimotor input current. The transformed points acquirement module contains eight digital TC neurons that are implemented in a parallel hardware topology. The updating module of UKF receives the observed membrane potentials using an ADC device. The estimated hidden properties are output from the prediction module of UKF to the peripheral DAC device. Since second-order UKF algorithm is employed and the estimated neuron model has three state variables and one estimated parameter, eight computational modules are demanded.

### The hardware-oriented CETC neuron model and its dynamical characteristics

In this study, a conductance-based TC neuron model is considered for the establishment of the RTDE system, with the gating variable *m* evolving on a much faster time-scale than variable *V*. This allows for model reduction and simplification, because *m* will approach the asymptotic value *m*_∞_ very quickly. Thus, *m* can be replaced by *m*_∞_ in sodium ion channel dynamics that enter into the voltage equation. Further, the potassium activation variable *n* is replaced by 1-*h* and TC relay neuron model is reduced to a three-dimensional model. The equation of the membrane potential *V* can be expressed as:


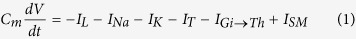


where *I*_*L*_, *I*_*Na*_, *I*_*T*_ and *I*_*K*_ are leak, sodium, low-threshold calcium and potassium spiking currents respectively. *I*_*Gi→Th*_ is the synaptic current from the globus pallidus internus (GPi) neuron to the TC relay neuron. *I*_*SM*_stands for sensorimotor input to the thalamus and takes the form


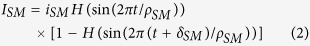


where *H* is the Heaviside step function, such that *H*(*x*) = 0 if *x* < 0 and *H*(*x*) = 1 if *x* > 0. Besides, *ρ*_SM_ is the period of *I*_SM_, *δ*_SM_ is the duration, and *i*_SM_ is the amplitude of positive input. The mathematical model of the TC relay neuron is based on the studies by Terman *et al*.^6^. The membrane capacitance *C*_*m*_ is unity. The two gating variables, which are the hidden properties of the TC relay cell, are described as


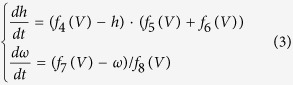


where the functions *f*_*4*_(*V*) = *h*_∞_(*V*), *f*_*5*_(*V*) = *a*_*h*_(*V*), *f*_*6*_(*V*) = *b*_*h*_(*V*), *f*_*7*_(*V*) = *ω*_∞_(*V*) and *f*_*8*_(*V*) = 1/*τ*_*ω*_(*V*). The variable “*h*” is the gating variable that represents the probability that an inactivation gate for sodium ions is open in the TC relay cell, and “*ω*” represents the gating variable in the T-type calcium ionic channel. The gating variable is the variable that can switch the channels between open and closed states in a neuron model. The ionic currents are defined as


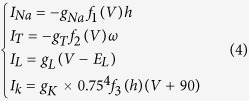


where the corresponding functions *f*_*1*_(*V*) = 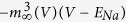, *f*_*2*_(*V*) = 
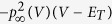
 and *f*_*3*_(*V*) = (1 − *h*)^4^. All of the nonlinear functions including *h*_∞_(*V*), *m*_∞_(*V*), *p*_∞_(*V*), *ω*_∞_(*V*), *a*_*h*_(*V*), *b*_*h*_(*V*) and *τ*_*ω*_(*V*) are given in the Supplementary information A. This model is called the original model in this study.

The nonlinearity in the original neuron model is a big challenge to achieve a cost-efficient hardware implementation of the biological conductance-based neuron model. The conventional method to implementing the nonlinear parts of the biological neuron model is based on the look-up table (LUT), which requires massive hardware resource. In order to further improve the computational efficiency and reduce the implementation cost in the RTDE system, a CETC neuron model is proposed to directly address this issue. In the CETC neuron model, the nonlinear functions are approximated with linear functions with the form:


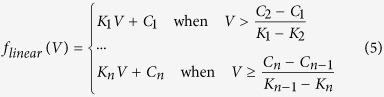


where *K*_*i*_ and *C*_*i*_ are the slope and intercept of each of linear functions respectively (*i* = 1, 2, ..., n). The modified functions are shown in Supplementary Figure S1. Since multipliers are extravagant hardware resource in the FPGA design, the values of *k*_*i*_ should be selected for the implementation only by “logic shift” and “add”. The coefficients of the linearized approximation are then determined (see Supplementary Table S1). Three items are important for determining the approximations of the original functions: (1) the piecewise linear approximations should have a high degree of fit, (2) the accuracy of the ionic currents should not adversely impact the dynamics of the neuron model, and (3) important features of the neural dynamics should be sustained after the piecewise linear approximations. The error criteria are defined in equations 6, 7, 8. The first cost function for error assessment is defined as:


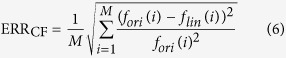


where *M* is the total number of sample points. The variable *f*_lin_ is the linearized approximation and *f*_ori_ is the original function. The normalized cost function for error assessment is given by


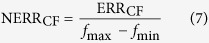


where *f*_max_ is the maximum value of the modified function, and *f*_min_ is the minimum value.

Another important measure in model error evaluations is MAE defined as


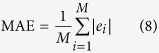


where the absolute error |*e*_*i*_| = |*f*_lin*i*_−*f*_ori*i*_|. The number of sample points *M* for each function is *M* = 1000 (see Supplementary Table S2). According to the results, the proposed CETC model has ideal precision with the mean ERR_CF_ = 0.0128 and MAE = 0.0696 for the CETC model. The NERR_CF_% is 1.9932% in comparison with the original model.

**Figure 2:**
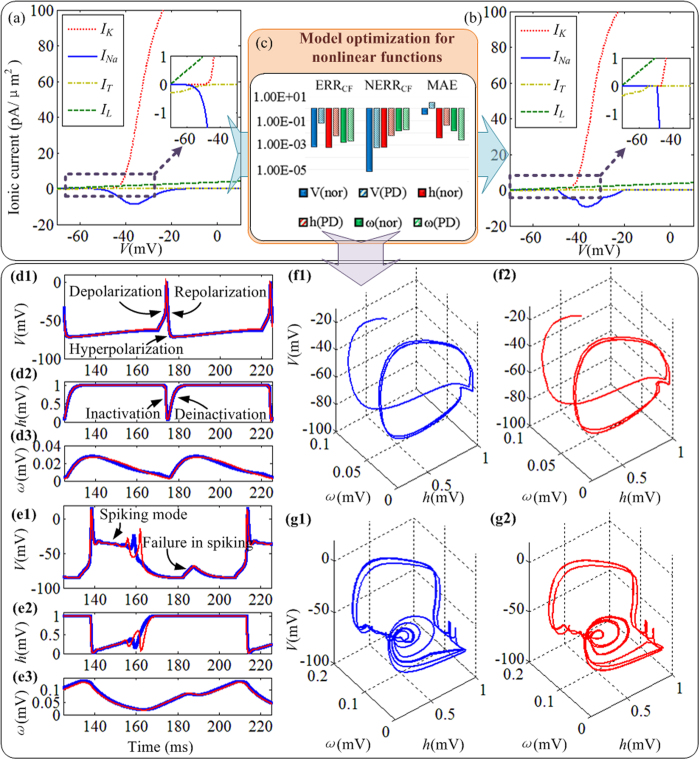
Ionic currents of TC relay neuron. (**a**) Ionic currents of the original model. (**b**) Ionic currents of the CETC model. (**c**) Error analysis of the dynamics of the CETC model. The notation “nor” in the bracket represents dynamics under the normal state, and “PD” refers to dynamics under the Parkinsonian state. All of the three variables in the proposed model are investigated. (**d**) Comparison of the output under the normal state. The blue line represents the original model and the red line represents the CETC model. (**e**) Comparison of the output under Parkinsonian state. The blue line represents the original model and the CETC model is represented by red line. (**f**) Stereoscopic image of a spiking trajectory of the TC relay neuron model under the normal state in the three-dimensional phase space (*V, h, ω*). The blue lines represent the dynamics of the original model in (**f1**), and the red lines represent the dynamics of the CETC model in (**f2**). (**g**) Stereoscopic image of a spiking trajectory of the TC relay neuron model under the Parkinsonian state in the three-dimensional phase space (*V, h, ω*). The blue lines represent the dynamics of the original model in (**g1**), and the red lines represent the dynamics of the CETC model in (**g2**).

To evaluate the neural dynamics of the CETC neuron model, the investigation of the ionic current provides an insight into the level of similarity through comparisons with the original model as shown in Fig. 2. The ionic currents are regarded as a function of voltage and the steady-state currents are calculated with the slow variables equal to their steady-state voltage values for fixed voltages. As shown in Fig. 2(a) and (b), it can be seen that the ionic currents of the CETC model are consistent with the original model. Error analysis of the dynamics of the CETC neuron model is considered in the proposed study, which is depicted in Fig. 2(c). We select ten thousand sampling points to obtain considerably reliable values of the errors. The Parkinsonian dynamics have regular bursting with rebound firing under a single stimulation, and all three of the error criteria are higher than those under the normal state except for the variable “*ω*” under the Parkinsonian state. Figure 2(c) indicates that the CETC model has an acceptable accuracy with a reliable dynamics.

Comparisons between the three variables of the original model and CETC model under the normal and Parkinsonian states are given in Fig. 2(d) and (e) respectively, which reveal that the CETC model can accurately reproduce the thalamocortical dynamics. The difference in the neuron model between the normal and Parkinsonian states is the value of the input current “*I*_*gi→th*_”, which is described in previous studies by Terman *et al*.^6^. A stereoscopic image is used to plot the firing trajectory of the TC relay neuron. Figure 2(f) and (g) show the stereoscopic images of a spiking and bursting trajectories of the original and CETC models in the three-dimensional phase space (*V, h, ω*). When the firing mode of the TC neuron is regular spiking, the trajectory of the neuron is a limit cycle. When the TC neuron bursts, the bursting trajectory slides along the bold half-parabola based on the locus of stable equilibrium with the variable *ω* slowly decreasing. The dynamics of the CETC and original models are consistent as shown in Fig. 2.

**Figure 3:**
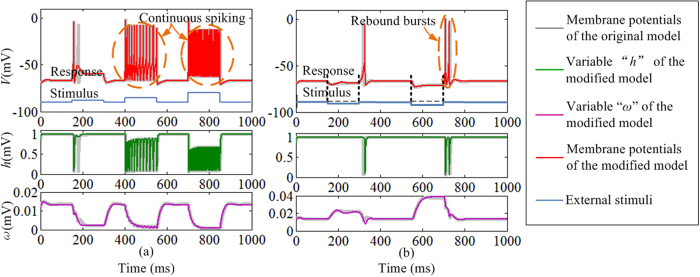
Comparison of original and CETC models to periodic stimulation. (**a**) The dynamical response of TC relay neuron to depolarizing input pulses. (**b**) The dynamical response of TC relay neuron to hyperpolarizing input pulses. The TC dynamical responses of the original model are plotted using grey lines, and the responses of the three variables “*V*”, “*h*” and “*ω*” in the CETC model are depicted in red, green and purple respectively.

The dynamical responses of the CETC neuron model under external stimulation are shown in Fig. 3. The TC neurons cannot fire spontaneously, and Fig. 3(a) shows that the TC neurons respond to positive depolarizing currents with continuous spikes with larger applied currents yielding faster responses. Figure 3(b) demonstrates that the TC neurons fire with strong rebound bursts following release under sustained negative hyperpolarizing currents and stronger rebound occurs with larger hyperpolarizing inputs. The TC neuron will faithfully follow a periodic external stimuli input over a wide range of input amplitude and frequency. The results reveal that the dynamical response of the CETC model is the same with original models. The original three-dimensional model using the conventional LUT-based method has been introduced by Yang *et al*.^34^. The resource cost can be significantly reduced thanks to the piecewise linear approximation approach and by replacing multipliers with barrel shifters and adders (see Supplementary Table S3). A barrel shifter is a digital circuit that can shift a data word by a certain number of bits using only pure combinatorial logic instead of any sequential logic. Results in Figs 2 and 3 show that the CETC model can both maintain the biological dynamics and reduce the hardware resource cost, which enables the RTDE system implementation and broadens the applications in the neuromorphic engineering.

### The application of the UKF in the CETC neuron model

In order to predict the hidden properties of the TC relay neuron, the UKF algorithm is required to be implemented into the TC neuron model. The TC neuron model is not just used to test the proposed system as the observation. It will be also implemented in the RTDE system to estimate the hidden properties. The procedure of the RTDE system and the task of each module in the system are described as follows:

#### The prediction module of UKF

For an *N*-dimensional estimated state *x*, we choose the sigma points from the mean value 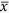 and covariance *P*_*xx*_ as:


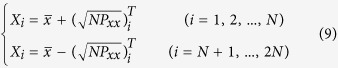


where *P*_*xx*_ is the estimated covariance matrix and 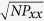 is the matrix square root. Sigma points are the sample points at the boundaries of a covariance ellipsoid. This procedure is implemented in the prediction module of UKF in the proposed RTDE system.

#### Transformed points acquirement module

The function *G* is applied to the sigma points with results 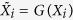 (*i* = 1, 2… 2*N*). The observation of the new state is represented by 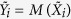. In the RTDE system, the nonlinear functions *G*(*X*) and *M*(*X*) use the CETC neuron model to yield transformed points and the observations, which are implemented in the transformed points acquirement module. To implement the UKF into the neuron model, the augmented state vector *x* as a *N* = *p* + *n* dimension vector composed of *p* parameters and *n* dynamic variables. In this paper, in order to estimate the synaptic current from GPi neurons to TC neurons, the external applied current *I*_*ext*_ is considered as a time-varying parameter and inserted into the state vector, which consists of the synaptic current *I*_*Gi→Th*_ and the sensorimotor input *I*_*SM*_. Thus, the process equations of the Kalman filter would be:


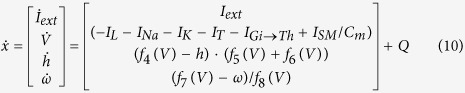


Since the only measured variable is the membrane potential *V* of the TC relay neuron, the measurement equations would be:


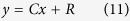


where C = [0 1 0 0]. By augmenting the observed state variables with system parameters and unobserved state variables, the UKF can track and estimate both the system parameter and unobserved variables.

#### The updating module of UKF

The updating module of UKF in the RTDE system assimilates noisy measurements to update the system state and covariance. The mean values are defined as


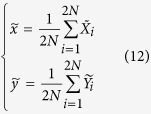


which stands for the *a priori* state estimate and *a priori* measurement estimate respectively. The *a priori* covariance of the ensemble members is defined as follows:


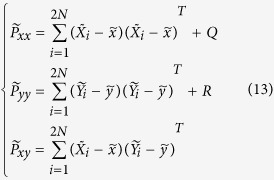


where *Q* and *R* stand for the covariance matrix of the process noise and observation noise respectively. The current state and error covariance are updated by the *a posteriori* quantities 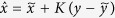 and 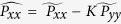 respectively. 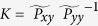
represents the Kalman gain matrix and *y* denotes the measurement value. The observation variable 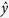 is updated by 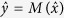. The updated 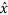 and 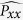 will be used for the next iteration.

### Estimation results of the RTDE system

**Figure 4:**
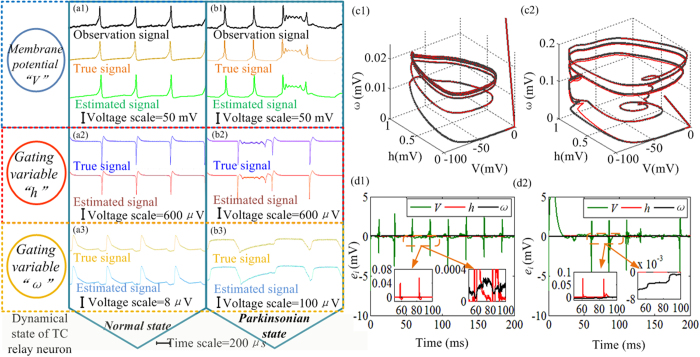
The oscilloscope photographs of the experimental results and its analysis. The oscilloscope photographs shows the actual dynamical behaviors of the TC neuron and the estimation results of the RTDE system in the normal and Parkinsonian states (time scale = 200 μs). In the RTDE system the hardware clock frequency is divided due to the hardware timing constraints and the clock division factor is 20 using a PLL block, so that the system timing requirement is met. The oscilloscope is used to display the experimental results and in high-resolution acquisition mode. (a1) TC neuron membrane potential in the normal state. (a2) The variable “*h*” of the TC neuron in the normal state. (a3) The variable “*ω*” of the CETC model in the normal state. (b1) TC neuron membrane potential in the Parkinsonian state. (b2) The variable “h” of the TC neuron in the Parkinsonian state. (b3) The variable “*ω*” of the TC neuron model in the Parkinsonian state. (c1) Stereoscopic image of a spiking trajectory of the TC relay neuron under the normal state in the three-dimensional phase space (*V, h, ω*). (c2) Stereoscopic image of a spiking trajectory of the TC relay neuron model under the Parkinsonian state in the three-dimensional phase space (*V, h, ω*). (d1) Absolute error of the estimation results under the normal state. (d2) Absolute error of the estimation results under the Parkinsonian state.

In the proposed study, we estimated the thalamocortical hidden properties within the UKF framework by assimilating the membrane potentials. The RTDE system uses FPGA-based UKF to reconstruct the hidden properties of the TC relay neuron from measured membrane potentials of a digital neuron, which is implemented based on the CETC neuron model. In Figs 2 and 3, the dynamical characteristics of the CETC neuron model have been proven to be consistent with the original TC relay neuron model presented by Terman *et al*. in 2004. Terman’s model is based on whole cell voltage-clamp recoding obtained from acutely dissociated thalamic relay neurons^6^,^41^,^42^,^43^. A measurement noise is added to the observation signals of the RTDE system to mimic the membrane potentials of the TC relay cells measured in the electrophysiological experiments^20^,^41^,^42^,^43^. By comparing the estimated values with the true values, we can validate the performance of the estimation. Figure 4 shows the estimation results of the dynamical behaviors of the TC neuron under the normal and Parkinsonian state respectively. The observation signal represents the noise-contaminated membrane potential that is used as the observation of the RTDE system. The performance of the UKF dynamical tracking strategy using the CETC model provides credible results. The proposed RTDE system is implemented on a Stratix-Ш EP3SE260 FPGA, which uses a total of 768 18-bit DSP block elements. A timing requirement needs to be translated into static timing constraints for an FPGA to be able to handle it. Thus, the timing constraints are applied to the hardware design and set up the clock period on the FPGA by using the phase-locked loop (PLL). A PLL is a feedback control system that automatically adjusts the phase of a locally generated signal to match the phase of an input signal. The clock division factor of the PLL, which defines the division ratio between the input clock frequency and the output clock frequency of the PLL, is set to be 20 in the proposed RTDE system for an appropriate system speed. The original model would require a greater than 50% increase in 18-bit DSP block making it impractical to be used in the UKF-based RTDE system. With the proposed CETC model, the UKF system can be implemented in the Stratix-Ш EP3SE260 FPGA with low resource cost.

Analysis of the experimental results is proposed to evaluate the estimation performance of the UKF. The estimation results are displayed using stereoscopic image of spiking trajectories under both normal and Parkinsonian states. In Fig. 4(c1) and (c2), the true and estimated values are plotted using black and red lines respectively. The absolute error *e*_*i*_ is calculated to investigate the performance of the estimation of the three TC neuron model variables. The values of “*e*_*i*_” under the normal and Parkinsonian states are depicted in Fig. 4(d1) and (d2) respectively. The error analysis reveals that high-performance estimation is obtained using the proposed RTDE system, thereby suggesting further application in electrophysiological experiments.

### Performance analysis for the RTDE system

In order to explore the effects of the process noise and observation noise covariance matrices *Q* and *R* on the estimation results and evaluate the estimation error of the algorithm, we use the cost function for RMSE, which is defined as


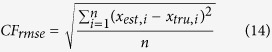


where *n* is the number of estimated points, and *x*_*est,i*_ and *x*_*tru,i*_ stand for the estimated (*est*) and the true values (*tru*), respectively.

**Figure 5:**
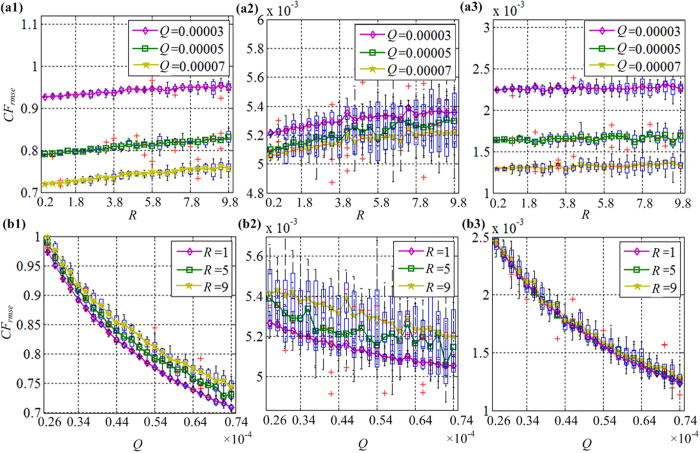
Effects of the covariance matrices for process noise *Q* and observation noise *R* on the cost function *CF*_*rmse*_. (**a1**) The effects of the observation noise *R* on the cost function *CF*_*rmse*_ of the membrane potential *V*. (**a2**) The effects of the observation noise *R* on the cost function *CF*_*rmse*_ of the slow variable *h*. (**a3**) The effects of the observation noise *R* on the cost function *CF*_*rmse*_ of the slow variable *ω*. (**b1**) The effects of the process noise *Q* on the cost function *CF*_*rmse*_ of the membrane potential *V*. (**b2**) The effects of the process noise *Q* on the cost function *CF*_*rmse*_ of the slow variable *h*. (**b3**) The effects of the process noise *Q* on the cost function *CF*_*rmse*_ of the slow variable *ω*.

The boxplots in Fig. 5 show the points, L-estimators, interquartile range, midhinge, range, mid-range and trimean. The estimation error of the membrane potentials increases with increasing observation noise *R*, or with the decreasing process noise *Q*, Fig. 5(a1) and (b1). The noise has a significant effect on the estimation error of the variable *h*, Fig. 5(a2) and (b2), similar to the effects of the noise on the variable *V.* Figure 5(a3) and (b3) shows that the observation noise *R* does not have a large effect on the estimation error of the slow variable *ω*. The time to reach steady-state will be longer as *Q* decreases, or with increasing *R*, so there exists a conflict between the estimation error and the time to steady-state. In the proposed design, we choose the UKF parameters *Q* = 0.00005 and *R* = 5.

**Figure 6:**
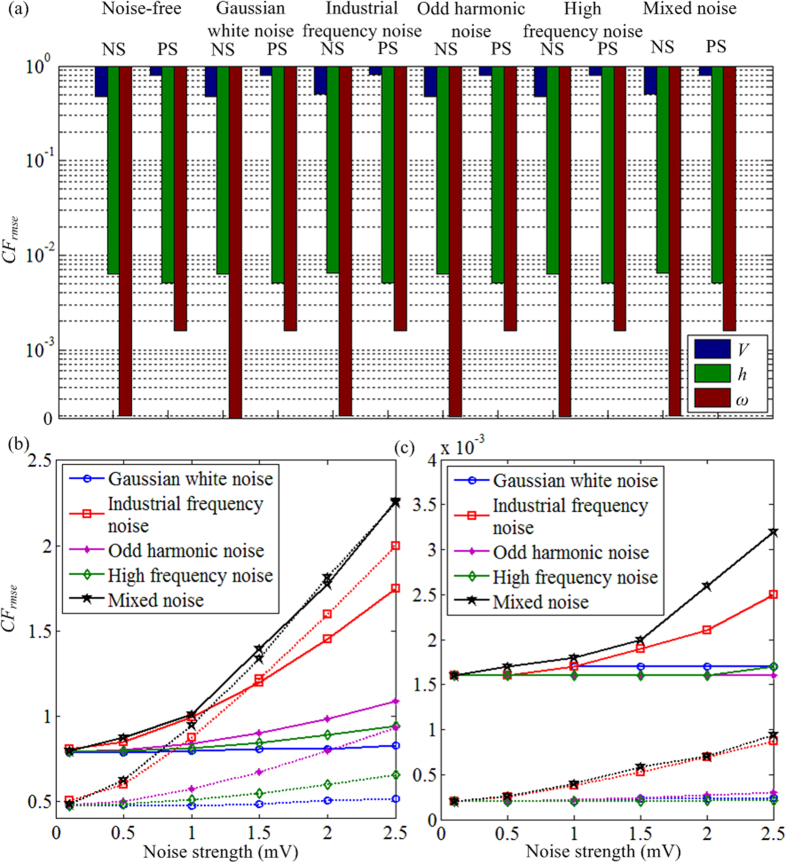
The investigation of the effects of noises on the estimation performance. (**a**) The effects of different kinds of noises on the estimation results under both the normal and the Parkinsonian state. NS and PS represent the normal and Parkinsonian states respectively. The errors of the three estimated variables are represented by the blue, green and brown columns respectively. (**b**) The effects of the noise strength on the estimation performance of the membrane potential. The dotted and solid lines represent the normal and the Parkinsonian states respectively. (**c**) The effects of the noise strength on the estimation performance of the hidden properties under the Parkinsonian state. The dotted and solid lines represent the normal and the Parkinsonian states respectively.

In order to investigate the estimation performance of the proposed RTDE system, we added different kinds of noises in the model data to mimic realistic measurement environments. As shown in Fig. 6(a), *CF*_*rmse*_ for noiseless observation is close to zero. Then we add the noise to mimic the measurements of the membrane potentials of the TC relay cells^20^,^41^,^42^,^43^,^44^. In Fig. 6(a), different kinds of noise are added to the observation. The noise includes the Gaussian white noise, industrial frequency noise (50 Hz), odd harmonic noise (150 Hz), high frequency noise (350 Hz) and mixed noise. The mixed noise is the commixture of the Gaussian white noise, industrial frequency noise, odd harmonic noise and high frequency noise. When the observation is noise-corrupted, the reconstructed hidden variables still have a good approximation to their true values. Besides, we investigate the effects of the noise strength on the estimation performance of the membrane potentials as shown in Fig. 6(b). Figure 6(c) shows the effects of the noise strength on the estimation error for the thalamocortical hidden properties. Both the normal and the Parkinsonian states have been considered. The effects of Gaussian white noise, high frequency noise and odd harmonic noise on the estimation performance are limited to a low level, as reflected by a small value of *CF*_*rmse*_. However, the estimation error under the industrial frequency noise increases significantly with the increment of the noise strength. The error induced by the mixed noise is larger than the errors under the other four kinds of noise and will increase more intensively with the noise strength increasing. The industrial frequency noise is the major factor of the greater error of the estimation performance under the mixed noise in both the normal and the Parkinsonian states. Fortunately, a notch filter can be used in the practical application to remove the industrial frequency noise. Thus, the errors of the estimation results for both the membrane potential and the hidden properties can still be quite small even with big noise strength, which suggests a good estimation performance of the proposed system to deal with the complex measurement environment such as electrophysiological experiments.

**Figure 7:**
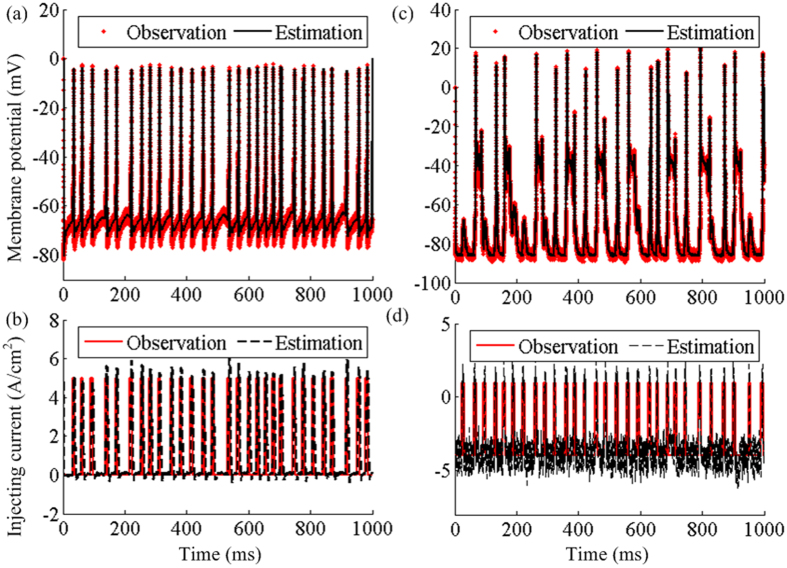
The double-blind experiment to test the estimation performance. (**a**) The estimation performance of the thalamocortical response under the normal state. (**b**) The estimation performance of the injecting current into the TC neuron under the normal state. (**c**) The estimation performance of the thalamocortical dynamics under the Parkinsonian state. (**d**) The estimation performance of the injecting current into the TC neuron under the Parkinsonian state.

Moreover, another main difference between the model-based data and the real data is the uncertainty of the unknown parameters in the real data. In order to explore the effectiveness of the UKF for a realistic system, a set of real data is of great importance. However, the major difference between the model-based data and the real data is reflected in the uncertainty of the unknown parameters in the real data. This uncertainty may increase the difficulties in dynamical estimation of the real data. In order to investigate the estimation performance on the real data, a double-blind experiment is introduced in our work. The double-blind experiment is that the implementation of the UKF-based system is not dependent on any parameters of the TC neurons, and meanwhile the voltage series of TC neurons is also generated independent on any parameters of the RTDE system. In the double-blind experiment, the parameters used in the model in the RTDE system are not the same with the observation and all the parameters of the observation are unknown. Since in a dynamic clamp experiment the injected current into a neuron is known, we can apply an external current to replace a sum of the synaptic current *I*_*Gi→Th*_and the sensorimotor input current *I*_*SM*_, depicted by the solid red lines as shown in Fig. 7(b) and (d). The sensorimotor input current *I*_*SM*_ takes the form of a series of monophasic current pulses whose amplitude is selected as 5pA/μm^2^ and duration is 5 ms. The instantaneous frequency of the input current follow a gamma distribution with an average rate of 30 Hz and a variation coefficient of 0.2, which induces the current *I*_*SM*_ to simulate the non-regular nature of the input current from the cortex to the thalamus^45^. By choosing *R* = 1, *Q* = 0.005 for the normal state and *R* = 0.5, *Q* = 0.5 for the Parkinsonian state, the estimation works well as shown in Fig. 7(a) and (c). It is worth noting that only the TC membrane potentials are observable in the proposed double-blind study. In order to evaluate the estimation performance, the injecting currents are also estimated and the estimation results are shown as dotted black lines in Fig. 7(b) and (d). We noticed that there exist fluctuations in the reconstructed injecting currents, which is because the mapping functions for sigma points given by equation 10 may varied from a real TC cell. The fluctuations may be resulted from the lack of frequency adaption current in the model using in the design of UKF. Since the focus of this study was to estimate the complex thalamocortical hidden properties using the simple model, we have ignored this feature in the model in this study. However, from the viewpoint of the estimation results of the hidden slow variable, although the model in the UKF is simple, the estimation still performs very well for observation data without known parameters. Thus, it can be concluded that the proposed UKF system is appropriate to the estimation of the underlying information from a real recording.

## Discussion

The Parkinsonian state is characterized with the synchronized bursting phenomenon of the basal ganglia network. In this study, we propose a method along with techniques to obtain hidden properties of the TC relay neuron to investigate the mechanism of PD. To the best of our knowledge, real-time implementation of the dynamical estimation for a TC relay neuron has been rarely reported until now. We tested the implementable hardware system using the reproduced biological firing patterns with noise from a digital TC neuron. Experimental results reveal that the proposed system can effectively estimate the hidden properties of the digital TC neuron with high precision. However, a limitation of the proposed work is the lack of the validation using real data. Thus, in order to further explore the effectiveness of the RTDE system for a realistic neuron, a double-blind experiment is introduced in our work. It is shown that the estimation still performs well. In the future work, we will focus on the applications of our RTDE system, especially for the application in the electrophysiological recordings. This result opens a pathway for the future design of neural dynamical estimation about the TC relay neuron by overcoming the high hardware cost, scalability and computational efficiency challenges, which is meaningful for the investigation and neuromodulation of the pathological states of the movement disorders. Besides, the proposed RTDE system is significantly beneficial for the performance enhancement and application extension of the conventional dynamic clamp system, which will be helpful for the revelation of new aspects and marvels of neural system dynamics.

In the implementation of the RTDE system, the UKF algorithm is required to be implemented into the neuron model to obtain transformed points and observations. A critical challenge to overcome for the real-time estimation system lies in the implementation of the complicated TC model. Major limitations of the paralleled model implementation include the number of available fast multipliers and the random access memory (RAM) resources on a single FPGA chip^33^,^34^. We propose a novel CETC neuron model to replace the nonlinear functions of the high-dimensional TC neuron model with relevant dynamics for the high-performance digital implementation. The experimental results showed that the CETC model requires reduced hardware resources and accurately reproduces the thalamocortical dynamical characteristics in both the normal and Parkinsonian states. Numerous studies have proposed FPGA-based implementation of the realistic neural networks with different hardware structures and methods for the real-time emulations of the large-scale networks^46^,^47^,^48^,^49^. The real-time emulations of the large-scale neural networks are of vital significance to understand how the brain transfers, decodes and processes information^50^,^51^. The CETC model with lower hardware overhead is useful for establishing the large-scale thalamus network, which is meaningful for further investigations of the basal ganglia and related movement disorders.

Although researchers have attempted to obtain better performance of control strategies using various filtering algorithms, successful applications are limited by the lack of sufficient computational capacity^22^. The ability to implement a system-on-a-chip control platform using the RTDE system in NCE studies is a key advantage of the proposed technique. The closed-loop control strategy of the TC relay neuron based on FPGA has also been implemented; however they cannot be used in the practical applications due to its limitations in estimating the hidden properties^34^. Using the proposed RTDE system, the hidden properties can be used in the closed-loop hidden-variable-based (HVB) control to provide a significant enhancement of control performance in comparison with open-loop control and closed-loop membrane-potential-based (MPB) control (see Supplementary information C and Supplementary Fig. S2). Moreover, previous experiments with dynamic clamp techniques have focused on some kinds of diseases including Huntington’s disease, epilepsy and Alzheimer’s disease^37^,^38^,^39^. Some neuromodulation studies have also employed the dynamic clamp system to investigate the effects and mechanisms of the neuromodulation on the neural system^19^,^40^. Thus, the proposed technique can be useful in the explorations for other kinds of diseases by replacing the neurological model in the proposed framework, and can improve the effects of the neuromodulation approach.

Another important application of the proposed estimation system is the decoding process in BMI projects^20^,^22^,^52^. The UKF algorithm of Li *et al*.^22^ and the kernel autoregressive moving average algorithm of Shpigelman *et al*.^53^ have applied non-linear models of neural tuning in closed-loop BMI, which paves the way for the application of nonlinear filtering algorithms in BMI applications. This work, which combines the estimation algorithm with the neuron model using real-time measurements from membrane signals, will be meaningful for the further development of the BMI for thalamus decoding. The problem in the application of the proposed system in BMI project is the design of algorithms that convert neural signals into control signals for the force-feedback device, which remains to be solved in future studies.

In terms of the future clinical applications, the proposed real-time platform is particularly applicable in TC driven prosthetic devices due to its reliable performance. While our estimates contribute to the explorations of the mechanism of PD, the technique and approach developed in this paper is also expected to be easily applicable to a wide variety of other diseases characterized by rapidly developing neurodegenerative dynamics, such as epilepsy or other kinds of neurological disorders.

## Methods

### Implementation of the UKF-based RTDE system

**Figure 8:**
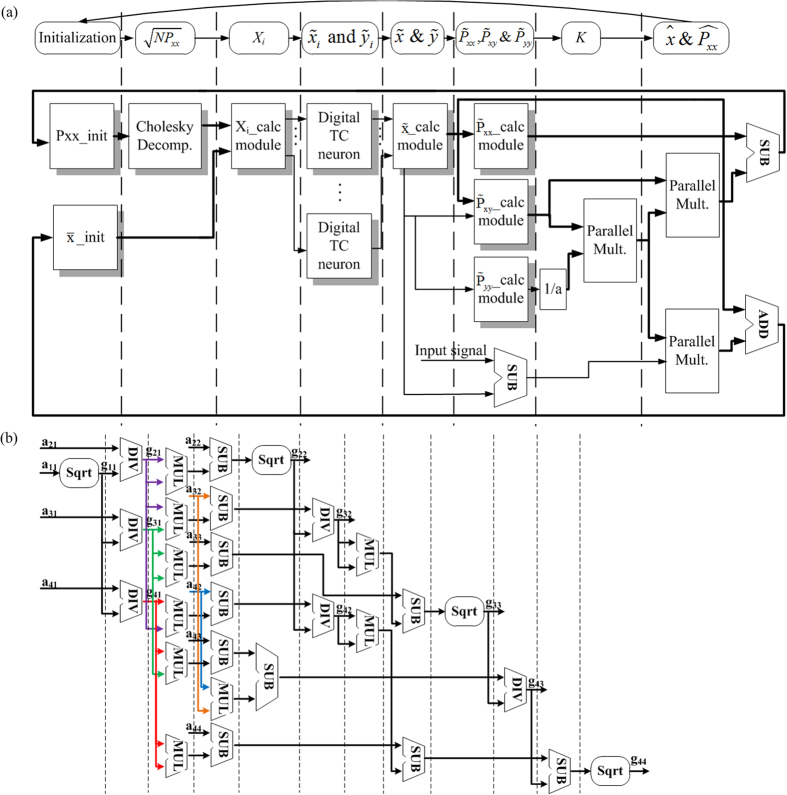
Digital topology of the proposed RTDE system. (**a**) Time-space diagram of data scheduling strategy of the RTDE system. The “Cholesky Decomp.” module implements the Cholesky decomposition. The “Parallel Mult.” module contains paralleled multiplication blocks. The “TC Calc.” module uses the digital implementation of the CETC model. (**b**) Digital implementation of the Cholesky algorithm.

Unlike previous studies in which only the decoding portion is designed on a FPGA, both decoding and encoding portions of the UKF algorithm are implemented in the proposed system, which facilitates the use of our system for online neural dynamical tracking or control using a portable device. The parallel system architecture is shown in Fig. 8(a). The “P_xx__init” and “
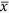
_init” module initializes of the covariance matrix P_xx_ and the mean value of the estimated state 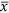. These two initialization modules will work in the first step of the proposed system and then work as registers. The Cholesky decomposition algorithm (see Supplementary information B) is implemented in the “Cholesky Decomp.” module. The “X_i__calc” module calculates the sigma points *X*_*i*_, and the eight “digital TC neuron” modules are used to propagate each sigma point from time step *t* to *t* + 1 yielding transformed points and the observations. The digital TC neuron uses the CETC neuron model to reduce the computational resources. The estimation of the new mean values based on transformed points is calculated in the “
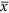
_calc” module and the estimation of the new covariance is implemented in the “
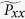
_calc”, “
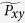
_calc” and “
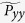
_calc” modules. The “Parallel Mult.” modules containing 2*N* multiplication blocks in parallel are used to compute the Kalman gain matrix *K*, updated mean and covariance matrix. The “1/a” module is designed to calculate the reciprocal value of “
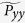
”. The “X_i__calc”, “
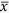
_calc”, “
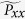
_calc”, “
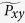
_calc” and “
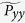
_calc” modules are implemented based on the UKF algorithm descript in equations 9, 12 and 13.

The digital structure of the Cholesky algorithm is shown in Fig. 8(b), which is a parallel architecture feasible for the FPGA-based implementation. In the “DIV” block for the division operation, the fixed-point divider can only output the quotient and remainder separately, so the numerator will be enlarged by a barrel shifter and divided by the denominator, and then the quotient is decreased by the same time for the division result with a barrel shifter. The fixed-point square root block can only output the integer square root, so the same approach is used in the “Sqrt” blocks. The “MUL” block represents the fixed-point multiplication operation and the “SUB” block stands for the fixed-point subtraction operation.

### Digital implementation of the TC neuron model

In the presented design, the Euler method is used to discretize the TC relay neuron model for simulations. Three blocks are used to compute the derivatives of the variables *V, h, ω*, and the modified ordinary differential equation for *V* is discretized into the equation:


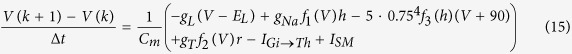


Similarly, the ordinary differential equations for two gating variables *h* and *ω* can be discretized as follows:


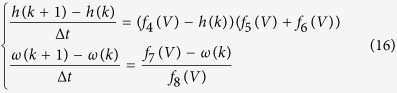


where *k* indexes the integration steps and Δ*t* is the time step in the Euler-based discretized equations. The parameter *C*_*m*_ in equation 16 is set to 1 pF/μm^2^ in this study. The synaptic current *I*_*Gi→Th*_ is determined based on the state of the neuron. The sensorimotor input *I*_*SM*_takes the form of a square wave with a period of 25 ms, duty ratio of 20% and amplitude of 5 mV.

**Figure 9:**
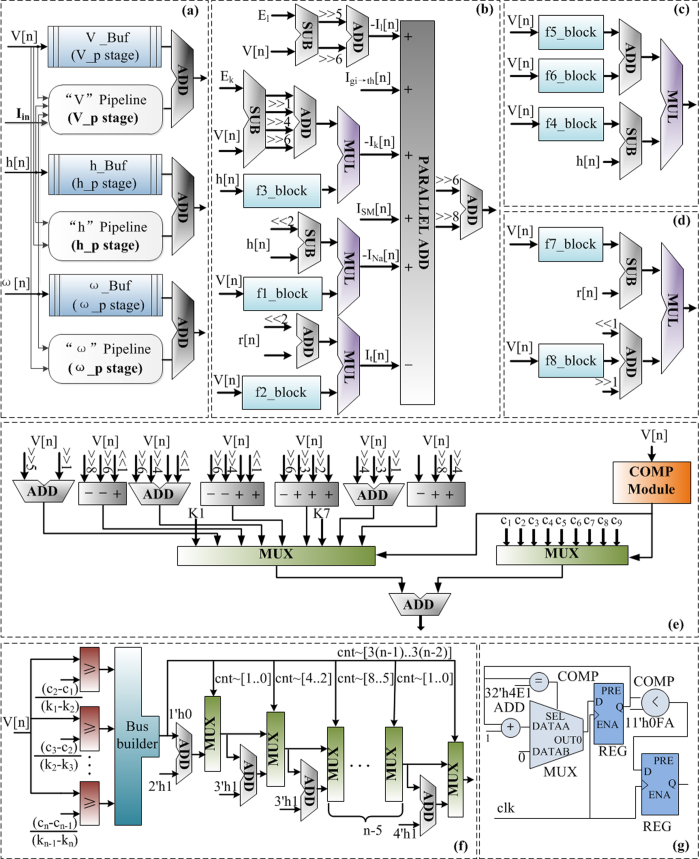
The digital topology of TC relay neuron model. (**a**) General overview of pipelining structure of the TC neuron model. (**b**) The *V* pipeline. (**c**) The *h* pipeline. (**d**) The *ω* pipeline. (**e**) The detailed digital topology of f1_block module. (**f**) The detailed digital topology of the COMP module in each fi_block module. (**g**) The digital implementation of the module of sensorimotor input current.

Pipelining technology is a significant approach to increase the throughput of the hardware system. There are three variables contained in the digital pipeline of TC neurons, so the hardware topology of the TC neuron has three pipelines and three buffers as shown in Fig. 9, which should be synchronized with each other at each clock pulse. In Fig. 9(a) the “*V*” pipeline module calculates the pipeline of variable “*V*” in V_p stages, which affects the computational cost in the digital implementation. The “h” and “*ω*” pipeline modules implement the pipelines of the variables “*h*” and “*ω*” in h_p and ω_p stages respectively. The V_Buf, h_Buf and *ω*_Buf store the variable values in the three pipelines separately. Figure 9(b,c and d) show the detailed digital structure of the “*V*”, “*h*” and “*ω*” pipeline modules respectively. The fi_block (*i* = 1, 2, …, 8) implements the linearized function in the CETC model. The “multiplication” operations with parameters are replaced by “add” and “shift” operations. Multiplication between two variables cannot be replaced by the “add” and “shift” operation, so the implementation of the proposed model must include some multipliers in its implementation.

Figure 9(e) shows the digital topology of the f1_block module for the computation of the “*f*_1_(*V*)” function. The hardware structures of other linearized functions are the same as the f1_block module with different coefficient values and segment number. The detailed digital structure of the COMP module is shown in Fig. 9(f), which employs a set of logic elements. The proposed method reduces the total block memory bits significantly. The number “n” in Fig. 9(f) is the segment number of the piecewise linear functions. The bus builder block is used to construct the output from inputs with a single bit. The module of sensorimotor input current is implemented based on FPGA as well to reproduce the cortical excitatory input current from the brain sensorimotor, and its detailed hardware structure is shown in Fig. 9(g). It is implemented using the digital logic elements of FPGA based on equation 2.

## Additional Information

**How to cite this article**: Yang, S. *et al*. Efficient implementation of a real-time estimation system for thalamocortical hidden Parkinsonian properties. *Sci. Rep.* **7**, 40152; doi: 10.1038/srep40152 (2017).

**Publisher's note:** Springer Nature remains neutral with regard to jurisdictional claims in published maps and institutional affiliations.

## Supplementary Material

Supplementary Information
