# Supplementary material for: Efficient implementation of a real-time estimation system for thalamocortical hidden Parkinsonian properties
Source: Sci Rep. 2017 Jan 9;7:40152. doi: 10.1038/srep40152 (PMC5220381; doi:10.1038/srep40152)
Supplement: Supplementary Information [file srep40152-s1.doc]

**Supplementary information**

**Efficient implementation of a real-time estimation system for thalamocortical hidden Parkinsonian properties**

Shuangming Yang^1^, Bin Deng^1^, Jiang Wang^1^, Huiyan Li^2^, Chen Liu^1,3^*, Chris Fietkiewicz^3^, Kenneth A. Loparo^3^

*^1^School of Electrical Engineering and Automation, Tianjin University, 300072, Tianjin, China*

*^2^ School of Automation and Electrical Engineering, Tianjin University of Technology and Educations, 300222, Tianjin, China*

^3^Department of Electrical Engineering and Computer Science, Case Western Reserve University, 44106, Cleveland, Ohio, USA

*Corresponding author. E-mail: [liuchen715@tju.edu.cn](mailto:liuchen715@tju.edu.cn); Fax/Tel: 86-22-27402293

**Contents**

1. Nonlinear functions in the TC neuron model 2
2. Cholesky decomposition 3
3. Comparison between MPB and HVB control strategies 5
4. Supplementary figures and tables 6
5. **Nonlinear functions in the TC neuron model.**

In this paper, we use a TC relay neuron model which is represented by membrane dynamics. The membrane potential *V* is described by the following equation:

$C_{m}\frac{dV}{dt}=-I_{L}-I_{Na}-I_{K}-I_{T}-I_{Gi\to Th}+I_{SM}$ (A.1)

where *I_Na_* and *I_K_* are the sodium and potassium ionic currents, *I_L_* represents the passive leak current, *I_T_* stands for the low-threshold T-type calcium current. *C_m_* represents the membrane capacitance and the synaptic input from GPi neuron to TC neuron is represented by *I_Gi→Th_*. In this model, there are some nonlinear functions in equations 3-4. The nonlinear function *p_∞_*(*V*) is the maximum permeability of the membrane for T-type calcium ions. The nonlinear functions *m*_∞_, *h*_∞_, *n*_∞_, *ω*_∞_ represent the voltage-sensitive steady-state functions and functions *τ*_m_, *τ*_h_, *τ*_n_, *τ*_ω_ represent the time constant of each channel. The nonlinear functions *m*_∞_, *h*_∞_, *ω*_∞_, *p*_∞_ and *τ_h_*, *τ_ω_* are defined as:

$$\left\{\begin{matrix} h_{\infty}\left(V\right)=\frac{1}{1+\exp\left(\frac{V+41}{4}\right)} \\ \omega_{\infty}\left(V\right)=\frac{1}{1+\exp\left(\frac{V+84}{4}\right)} \\ m_{\infty}\left(V\right)=\frac{1}{1+\exp\left(-\frac{V+37}{7}\right)}\text{ } \\ p_{\infty}\left(V\right)=\frac{1}{1+\exp\left(-\frac{V+60}{6.2}\right)} \\ \tau_{\omega}\left(V\right)=28+\exp\left(-\frac{V+25}{10.5}\right) \\ \tau_{h}\left(V\right)=\frac{1}{a_{h}\left(V\right)+b_{h}\left(V\right)} \end{matrix}\right.\text{ }\left(\text{A.2}\right)$$

Other ion channel variables are described as:

$\left\{\begin{matrix} a_{h}\left(V\right)=0.128\exp\left(-\frac{V+46}{18}\right) \\ b_{h}\left(V\right)=\frac{4}{1+\exp\left(-\frac{V+23}{5}\right)}\text{ } \end{matrix}\right.$ (A.3)

The TC relay neuron is considered to be at rest when its potential is around -65mv in the absence of inputs from both the GPi and the cortex. The inhibitory current from GPi as a hyperpolarization input current would induce a rebound burst, and the excitable current from the cortex as a depolarizing input current would elicit tonic spikes.

1. **Cholesky decomposition.**

The Cholesky decomposition of a positive definite matrix *Q* can be expressed as *Q*=*R*^T^*R*, in which *R* is an upper triangular matrix with positive diagonal elements, and, the result of the Cholesky decomposition is unique. There are important algorithms based on the Cholesky decomposition, such as the Cholesky-Banachiewicz and Cholesky-Crout algorithms. Suppose the covariance matrix to be decomposed is Q, which is defined as

$\left\{\begin{matrix} Q=\left[\begin{matrix} a_{11} & a_{21} & a_{31} & a_{41} \\ a_{21} & a_{22} & a_{32} & a_{42} \\ a_{31} & a_{32} & a_{33} & a_{43} \\ a_{41} & a_{42} & a_{43} & a_{44} \end{matrix}\right]=\text{R}^{\text{T}}\text{R} \\ \text{R}^{\text{T}}=\left[\begin{matrix} g_{11} & 0 & 0 & 0 \\ g_{21} & g_{22} & 0 & 0 \\ g_{31} & g_{32} & g_{33} & 0 \\ g_{41} & g_{42} & g_{43} & g_{44} \end{matrix}\right] \end{matrix}\right.\text{ }$ (B.1)

Then we have

$\begin{matrix} Q=R^{\text{T}}R=\left[\begin{matrix} g_{11} & 0 & 0 & 0 \\ g_{21} & g_{22} & 0 & 0 \\ g_{31} & g_{32} & g_{33} & 0 \\ g_{41} & g_{42} & g_{43} & g_{44} \end{matrix}\right]\left[\begin{matrix} g_{11} & g_{21} & g_{31} & g_{41} \\ 0 & g_{22} & g_{32} & g_{42} \\ 0 & 0 & g_{33} & g_{43} \\ 0 & 0 & 0 & g_{44} \end{matrix}\right] \\ =\left[\begin{matrix} g_{11}^{2} & g_{11}g_{21} & g_{11}g_{31} & g_{11}g_{41} \\ g_{11}g_{21} & g_{21}^{2}+g_{22}^{2} & g_{21}g_{31}+g_{22}g_{32} & g_{21}g_{41}+g_{22}g_{42} \\ g_{11}g_{31} & g_{21}g_{31}+g_{22}g_{32} & g_{31}^{2}+g_{32}^{2}+g_{33}^{2} & g_{31}g_{41}+g_{32}g_{42}+g_{33}g_{43} \\ g_{11}g_{41} & g_{21}g_{41}+g_{22}g_{42} & g_{31}g_{41}+g_{32}g_{42}+g_{33}g_{43} & g_{41}^{2}+g_{42}^{2}+g_{43}^{2}+g_{44}^{2} \end{matrix}\right] \end{matrix}$ (B.2)

Then we have the formulas:

$\left\{\begin{matrix} g_{j,j}=\sqrt{a_{j,j}-\sum_{k=1}^{j-1} g_{j,k}^{2}} \\ g_{i,j}=\frac{1}{L_{j,j}}\left(a_{i,j}-\sum_{k=1}^{j-1} g_{i,k}g_{j,k}\right),\text{ for }i>j \end{matrix}\right.$ (B.3)

The Cholesky-Banachiewicz algorithm is used to calculate the matrix row by row, and the Cholesky-Crout algorithm is used to calculate the matrix column by column. Then we have the decomposition result:

$\left\{\begin{matrix} g_{11}=\sqrt{a_{11}} \\ g_{21}=a_{21}/g_{11} \\ g_{31}=a_{31}/g_{11} \\ g_{22}=\sqrt{a_{22}-g_{21}^{2}} \\ g_{32}=\left(a_{32}-g_{31}\cdot g_{21}\right)/g_{22} \\ g_{41}=a_{41}/g_{11} \\ g_{33}=\sqrt{a_{33}-g_{31}^{2}-g_{32}^{2}} \\ g_{42}=\left(a_{42}-g_{41}\cdot g_{21}\right)/g_{22} \\ g_{43}=\left(a_{43}-g_{41}\cdot g_{31}-g_{42}\cdot g_{32}\right)/g_{33} \\ g_{44}=\sqrt{a_{44}-g_{41}^{2}-g_{42}^{2}-g_{43}^{2}} \end{matrix}\right.$ (B.4)

Using the Cholesky decomposition can improve the speed of matrix computations by a factor of two, or greater and because pivoting is not necessary, errors will also be reduced. Suppose that **A***x*=**b** and *y* represents the calculated solution, *y* solves the system of equations (**A**+**E**)*y*=**b** and

$\left\|\text{E}\right\|_{2}\leq c_{n}\varepsilon\left\|\text{A}\right\|_{2}$ (B.5)

where “|| ||_2_” denotes the matrix 2-norm, *c_n_* stands for a small constant based on the value of *n*, and *ε* represents the unit round-off. Square roots are used in the Cholesky decomposition, so the results after the square root are positive (the factorized matrix is required to be positive definite). However, if the matrix is ill-conditioned, the results can be negative due to round-off errors and the algorithm cannot continue processing. One of the solutions is to add a diagonal correction matrix to the coefficient matrix in order to guarantee positive-definiteness. Although the method may decrease the precision of the decomposition, it will enhance the stability of the decomposition.

1. **Comparison between MPB and HVB control strategies.**

The value of *rms* is determined by the following equation:

(C.1)

where *V_CSi_* represents the *i*^th^ value of the control signal and *N* is the total number of values. The standard deviations is described by the following equation:

$f_{s}=10\times\sqrt{\frac{1}{T}\int_{t_{0}}^{t} \left(V_{CS}\left(\tau\right)-V_{ess}\right)^{2}d\tau}$ (C.2)

where *f_s_* is the function that determines the standard deviations of the control signals and *T* represents the control period. Assuming that *V_ess_*=-15, the standard deviation *f_s_* of the control signals based on both the fast and slow variables are depicted in Fig. S2(c). The corresponding adjustment time is computed if the control signal can keep the error in the error band *Δ*=5%, see Fig. S2(d), where the slow-variable-based control strategy can shorten the adjustment time as well as reduce the signal power effectively. Therefore, using the proposed estimation system is helpful to track and extract the dynamics of the slow variable, which is then applied to the neural control system.

1. **Supplementary figures and tables.**


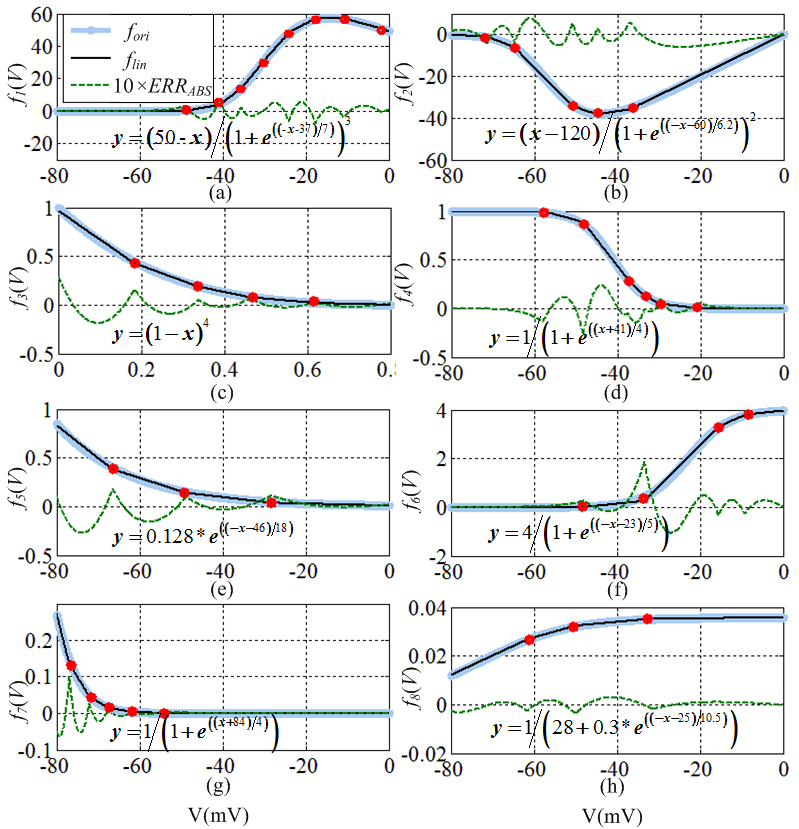


**Supplementary Figure S1. Modified functions in the neuron model.** Blue lines represents the original functions. Red dots are the knot points for the piecewise linear approximation and the segments connecting the red dots plotted by blue lines are the linear approximation segments for each linear function. Green lines describes ten times of the absolute error *ERR_ABS_*. We enlarge the value of absolute error tenfold to show the error more explicitly.


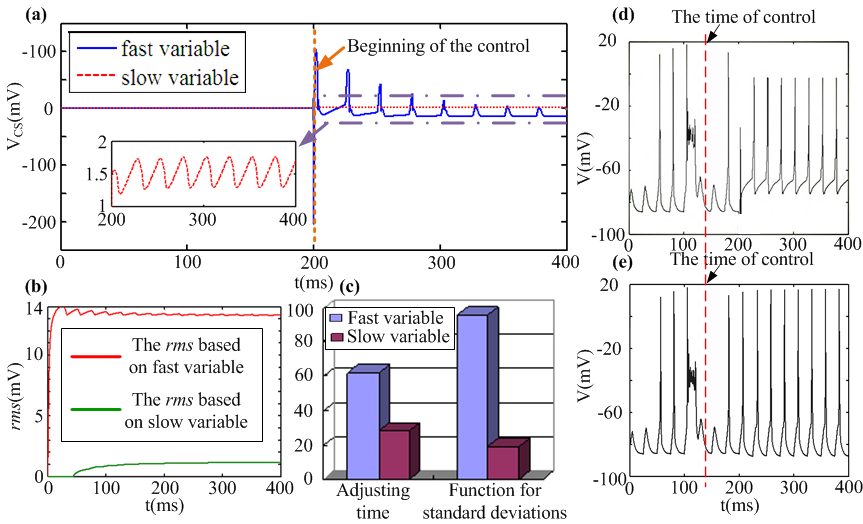


**Supplementary Figure S2. The comparison of the closed-loop control of the TC relay neuron using membrane-potential-based strategy and slow-variable-based strategy.** (a) The control signals of the closed-loop system. The “V_CS_” represents the voltage of the control signal. The blue solid line represents the control signal based on membrane potentials, and the red dotted line stands for the signal based on the slow variable. (b) The comparison between the values of *rms* based on fast and slow variables. (c) The comparison of the adjusting time and *x_rms_*, which describes the power of the control signals. (d) The control effect of the PID algorithm based on membrane potentials using the proposed RTDE system. (e) The control effect of the PID algorithm based on slow variable using the proposed RTDE system.

**Supplementary Table S1.**The values of coefficients in the piecewise linear model. The double dash represents the absence of the values in the corresponding function.

|  | *f_1_*(*V*) | *f_2_*(*V*) | *f_3_*(*V*) | *f_4_*(*V*) | *f_5_*(*V*) | *f_6_*(*V*) | *f_7_*(*V*) | *f_8_*(*V*) |
| --- | --- | --- | --- | --- | --- | --- | --- | --- |
| *K_1_* | 0 | -3/128 | -3.5 | 0 | -0.0752 | 0 | -23/512 | 1/2048 |
| *K_2_* | 15/32 | -13/32 | -2.75 | -3/256 | -17/512 | 9/512 | -0.0186 | 7.93×10^-4^ |
| *K_3_* | 507/256 | -9/4 | -25/16 | -7/128 | -7/512 | 11/64 | -7/1024 | 1/2048 |
| *K_4_* | 197/64 | -55/8 | -57/64 | -21/512 | -0.0049 | 79/1024 | -1/512 | 5/32768 |
| *K_5_* | 93/32 | -9/2 | -3/8 | -0.0225 | -1/1024 | 19/1024 | -1/2048 | 1/65536 |
| *K_6_* | 87/64 | -1 | -13/128 | -5/1024 | -- | -- | 0 | -- |
| *K_7_* | 0 | 3/4 | 0 | -1/32768 | -- | -- | -- | -- |
| *K_8_* | -11/16 | 33/32 | -- | -- | -- | -- | -- | -- |
| *K_9_* | -15/16 | -- | -- | -- | -- | -- | -- | -- |
| *C_1_* | 0 | -2.1 | 0.996 | 1 | -5.2683 | 0 | -3.32 | 0.0500 |
| *C_2_* | 22.92 | -32.55 | 0.94 | 0.32 | -1.8527 | 0.8473 | -1.292 | 0.0756 |
| *C_3_* | 84.50 | -162.5 | 0.715 | -1.751 | -0.5296 | 5.9545 | -0.447 | 0.0568 |
| *C_4_* | 123.55 | -461 | 0.488 | -1.24 | -0.1050 | 4.4959 | -0.1175 | 0.0400 |
| *C_5_* | 118.40 | -343 | 0.25 | -0.62 | 0.0080 | 3.9592 | -0.0265 | 0.0357 |
| *C_6_* | 81.09 | -187 | 0.0813 | -0.1 | -- | -- | 0 | -- |
| *C_7_* | 57.10 | -124 | 0 | 0 | -- | -- | -- | -- |
| *C_8_* | 49.60 | -120.96 | -- | -- | -- | -- | -- | -- |
| *C_9_* | 49.19 | -- | -- | -- | -- | -- | -- | -- |

**Supplementary Table S2.** Error evaluation of the modified model.

|  | ERR_CF_ | NERR_CF_% | MAE |
| --- | --- | --- | --- |
| *f_1_*(*V*) | 0.0219 | 0.0384 | 0.1691 |
| *f_2_*(*V*) | 0.0100 | 0.0261 | 0.3466 |
| *f_3_*(*V*) | 0.0048 | 0.4800 | 0.0050 |
| *f_4_*(*V*) | 0.0102 | 1.0200 | 0.0046 |
| *f_5_*(*V*) | 0.0031 | 3.1000 | 0.0069 |
| *f_6_*(*V*) | 0.0228 | 0.5700 | 0.0241 |
| *f_7_*(*V*) | 0.0293 | 10.700 | 0.0006 |
| *f_8_*(*V*) | 0.0003 | 0.0107 | 0.0001 |
| Mean value | 0.0128 | 1.9932 | 0.0696 |

**Supplementary Table S3.** Device utilization of the Stratix-Ш EP3SE260 Platform FPGA. Abbreviations: C. ALUTs (Combinational ALUTs), M. ALUTs (Memory ALUTs), DLC (Dedicated logic registers), Reg. (Register), TBMB (Total block memory bits), DSP B. (DSP block 18-bit elements), PLLs (Total PLLs).

|  |  | Original | Modified | UKF system |
| --- | --- | --- | --- | --- |
| Resource | Available | Resource utilization | | |
| C. ALUTs | 203520 | 1218 (1%) | 3554 (2%) | 42,295 (21%) |
| M. ALUTs | 101760 | 0 (0%) | 0 (0%) | 0 (0%) |
| DLC | 203520 | 132 (<1%) | 1004 (<1%) | 3130 (2%) |
| TBMB | 15,040,512 | 276,480 (2%) | 0 (0%) | 0 (0%) |
| DSP B. | 768 | 144 (19%) | 20 (3%) | 442 (58%) |
| PLLs | 8 | 1 (13%) | 1 (13%) | 1 (13%) |
